# Bothersome Back Exchange in MALDI Plume and Its Impact on Hydrogen/Deuterium Exchange Mass Spectrometry Analysis

**DOI:** 10.1002/jms.5108

**Published:** 2024-12-22

**Authors:** Xianwen Lou, Michel van Houtem, René P. M. Lafleur, Sandra M. C. Schoenmakers, Joost L. J. van Dongen, Anja R. A. Palmans

**Affiliations:** ^1^ Department of Chemical Engineering and Chemistry, Institute for Complex Molecular Systems Eindhoven University of Technology Eindhoven The Netherlands; ^2^ SyMO Chem B.V. Eindhoven The Netherlands

**Keywords:** back exchange, HDX, MALDI, MS, reactive plume

## Abstract

One critical issue in hydrogen/deuterium exchange mass spectrometry (HDX MS) analysis is the deleterious back exchange. Herein, we report that when matrix‐assisted laser desorption/ionization (MALDI) is used, the MALDI process itself can also cause significant back exchange. The back exchange occurred inside the reactive MALDI plume was investigated by depositing a fully deuterated sample prepared in D_2_O on top of a preloaded dried layer of matrix. A benzene‐1,3,5‐tricarboxamide (BTA) compound that can form supramolecular polymer in water and five peptides of angiotensin II (AT), pentaglycine (5G), pentaalanine (5A), cyclohexaglycine (C6G), and cyclohexaalanine (C6A) were selected as the testing compounds. Just like the situation in solution, the back exchange for the side chains and end groups is fast in the MALDI plume, while for the backbone amides, it is slow and dependent on the primary structure of the peptide. For the peptides tested, 5%–15% of D‐labels in the backbone amides can be lost during the MALDI process. This degree of back exchange, although not an unbearable problem for most HDX MS applications as 85%–95% of the informative labels would still survive, could seriously limit the use of MALDI in the HDX MS analysis of supramolecular assemblies. For these assemblies, the EX1‐like mechanism with two distinct distributions is common, and the back exchange could gravely distort or even merge the distinct isotopic distributions, which are the characteristic symbols of EX1.

## 1 Introduction

Hydrogen/deuterium exchange mass spectrometry (HDX MS) is a well‐established and powerful technique for the study of protein structures and dynamics [1, 2, 3, 4, 5, 6]. Recently, we have also used this technique to track the dynamics of supramolecular polymers in water [7, 8, 9, 10]. Supramolecular polymers in water represent an interesting class of materials, because their inherent dynamic conduct can be tuned to match the dynamic behavior of the supramolecular interactions found in living tissues [11]. In addition to electrospray ionization (ESI), which is usually the ionization method of choice for most applications, matrix‐assisted laser desorption/ionization (MALDI) has also been used in HDX MS because of its straightforward sample preparation, tolerance to chemical denaturants and buffers, and relatively simple mass spectra dominated by singly charged ions [12, 13, 14, 15].

A vital problem in HDX MS is the occurrence of back exchange after labelling, which will wash out the deuterium patterns imprinted on the labelled molecules. Obviously, for a successful analysis, back exchange must be prevented or carefully controlled. To this end, every step that might cause back exchange has to be examined systematically. Various approaches have been developed to maximize the preservation of the deuterium labels during protein digestion and high performance liquid chromatography (HPLC) separation [1, 2, 3, 4, 5, 6, 16, 17]. When MALDI is used, additional back exchange can arise during the preparation of a MALDI spot and during the desorption/ionization process initiated by laser irradiation. The back exchange occurred during the preparation of the MALDI spot can greatly be reduced by the combination of cooling down the sample solution and the matrix solution and using a chilled target plate [12, 13]. Surprisingly, however, the back exchange during the desorption/ionization process in HDX‐MS has not received significant attention.

It is well known that desorption/ionization in MALDI is a very complicated process which consists of a series of physicochemical events [18, 19, 20, 21]. The environment of the MALDI plume can be very reactive, containing matrix neutrals and ions, electrons, hydrogen atoms, and so forth [22]. In many cases, the final ions recorded in a MALDI spectrum are determined by the secondary reactions in this reactive dense plume [18, 19, 20, 21]. By using a delicately designed split sample holder with deuterated and non‐deuterated matrices, deuterium transfer was found to be a major pathway for MALDI gas phase ionization [23]. Obviously, all these reactions will very likely give rise to back exchanges in HDX MS analysis because the deuterium labels are usually labile and can be lost by collisions with various reactive species in the dense MALDI plume.

To assess the degree of back exchange generated solely by the gas‐phase reactions inside the MALDI plume, some fully deuterated samples dissolved in D_2_O were tested. Efforts were made to circumvent any back exchange before laser irradiation by carefully depositing the sample on top of a preloaded dried matrix layer [24]. As HDX‐MS is one of the primary tools used in our laboratory to probe the formation of supramolecular assemblies, we started with a benzene‐1,3,5‐tricarboxamide (BTA‐d_6_) compound, which is a well‐studied motif for one‐dimensional supramolecular assemblies [25]. Then, a number of peptides with and without the labile labels in the side chains or the end groups were purchased or synthesized to study the difference in the back exchange rates of the labels in the backbones, the side chains, and the end groups. Finally, the possible impact of the back exchange on the HDX MS analysis was investigated and discussed for protein/peptide samples and a BTA supramolecular polymer.

## 2 Experimental Section

### 2.1 Chemicals and Reagents

Benzene‐1,3,5‐tricarboxamide (BTA, C_69_H_129_O_18_N_3_) was synthesized as described previously [25]. Angiotensin II (AT), pentaglycine (5G), and pentaalanine (5A) were purchased from Sigma‐Aldrich (Zwijndrecht, the Netherlands). Cyclohexaglycine (C6G) and cyclohexaalanine (C6A) were synthesized using a patented method of AF Chemicals [26].

Three matrices were used in this study. *α*‐Cyano‐4‐hydroxycinnamic acid (CHCA) was obtained from Fluka (Zwijndrecht, the Netherlands), 2′,6′‐dihydroxyacetonephenone (DHAP) from Sigma‐Aldrich, and 2‐[(2E)‐3‐(4‐tert‐butylphenyl)‐2‐methylprop‐2‐enylidene]malononitrile (DCTB) was synthesized according to Ulmer et al. [27].

### 2.2 Analytical Protocols

The 500‐μM BTA solutions were prepared by dissolving weighted amounts of the compound in H_2_O or D_2_O, respectively. The mixtures were heated at 80°C for 15 min, followed by vortexing for 15 s, and then left to cool down and equilibrate at room temperature overnight. The HDX experiment was performed by diluting a BTA H_2_O solution 100 times in D_2_O. All the peptide solutions, except those of 5G and 5A, were prepared directly in H_2_O or D_2_O. To dissolve 5G and 5A, 0.5% of formic acid (FA) and FA‐d were added to H_2_O and D_2_O, respectively.

The MALDI spot was prepared by using a modified thin‐layer sample deposition method [24], in which a HDX sample solution was deposited on the surface of matrix preloaded on the MALDI plate to minimize the contact between the analyte and matrix before laser irradiation. D_2_O will not re‐dissolve the preloaded matrix preventing the mixing and co‐crystallization of the analyte with the matrix. The sample solutions and the MALDI plate were cooled to 0°C prior to sample deposition. Immediately after the deposition of the D_2_O solution on the matrix surface, the MALDI plate was quickly loaded into the vacuum chamber of the MALDI MS system to minimize the sample preparation time and to avoid the contact between a dried sample and air moisture.

The back exchange occurred inside the reactive MALDI plume was investigated by depositing a fully deuterated sample prepared in D_2_O on top of a preloaded dried layer of matrix.

For the back exchange experiments on the amide backbones, 100 μL of a fully deuterated solution of peptide in D_2_O precooled at 0°C was added into 2 mL H_2_O (with 0.1% of FA, pH = 2–3) also precooled at 0°C. To ensure that all the deuterons in the end groups and side chains had sufficiently been replaced by protons, the quenched solution was equilibrated for 2 min before being measured by ESI MS or MALDI MS.

### 2.3 Mass Spectrometry

The MALDI TOF MS measurements were performed with an Autoflex Speed (Bruker, Bremen, Germany) instrument. The accelerating voltage was held at 19 kV and the delay time at 130 ns. Mass spectra were acquired in the reflector positive ion mode by summing spectra from 500 random laser shots at an acquisition rate of 100 Hz.

The ESI‐MS measurements were carried out using a Xevo G2 QTof mass spectrometer (Waters, Wilmslow, UK) with a capillary voltage of 2.7 kV and a cone voltage of 20 V. The source temperature was set at 100°C, the desolvation temperature at 400°C, and the gas flow at 500 L/h. The sample solutions subjected to HDX were introduced into the mass spectrometer using a Harvard syringe pump (11 Plus, Harvard Apparatus) at a flow rate of 50 μL/min.

## 3 Results and Discussion

A persistent problem in HDX MS is the degradation of the labelled information due to back exchange. Thus, every process that could induce back exchange should be assessed critically. To evaluate the extent of back exchange resulting from the gas phase reactions in the MALDI plume, a fully deuterated sample in D_2_O was deposited on top of a preloaded dried matrix layer. Prior to the sample deposition, both the deuterated sample solution and the MALDI plate with preloaded dried matrix had been cooled to 0°C by placing them in ice for 1 h. Immediately after the sample deposition, the cold plate was quickly loaded into the MALDI vacuum chamber. In this way, back exchange before laser irradiation should be negligible, and therefore, the back exchange observed in these experiments should be directly attributed to the gas phase reactions during the MALDI process.

**FIGURE 1:**
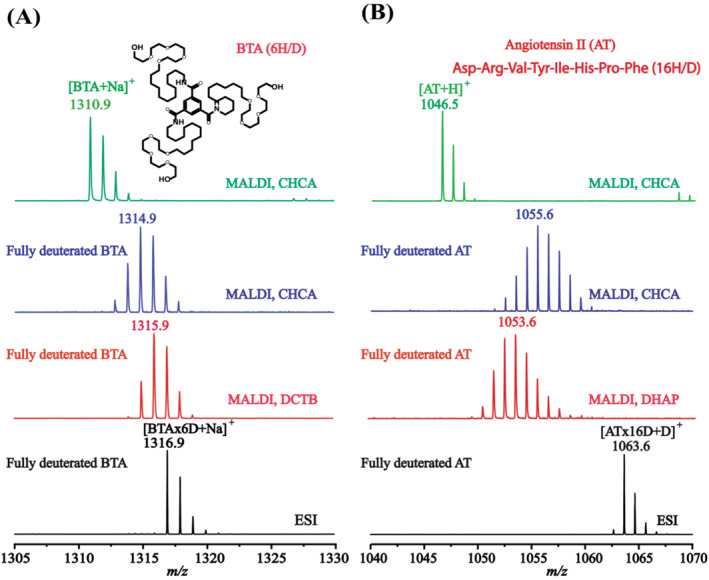
MALDI and ESI mass spectra of a benzene‐1,3,5‐tricarboxamide (BTA) and angiotensin II (AT). (A) Mass spectra for BTA, the top (spectrum in green) and the bottom (spectrum in black) are reference spectra of non‐deuterated BTA (BTAx0D in H_2_O) using CHCA matrix with MALDI and of fully deuterated BTA (BTAx6D in D_2_O) with ESI, respectively; the two middle ones are the MALDI spectra of the fully deuterated BTA (BTAx6D in D_2_O) with CHCA (spectrum in blue) and DCTB (spectrum in red), respectively. (B) Mass spectra for AT, the top (spectrum in green) and the bottom (spectrum in black) are reference spectra of non‐deuterated AT (ATx0D in H_2_O) using CHCA matrix with MALDI and of fully deuterated AT (ATx16D in D_2_O) with ESI, respectively; the two middle ones are the MALDI spectra of the fully deuterated AT (ATx16D in D_2_O) with CHCA (spectrum in blue) and DHAP (spectrum in red), respectively.

Figure 1A shows the MS spectra of a benzene‐1,3,5‐tricarboxamide (BTA) compound using CHCA or DCTB as the matrix. The BTA molecules can form supramolecular fibers in water, and HDX MS has been found to be a powerful tool to reveal the dynamics of the fibers [7]. A BTA molecule has three hydroxy groups at the periphery and three amide groups at the core. In a fully deuterated BTA molecule, these six labile hydrogens are all exchanged by deuteriums, which was confirmed by ESI‐MS (see the spectrum in black at the bottom of Figure 1A). The monoisotopic mass of [BTAx6D + Na]^+^ is 1316.9. However, serious back exchange was observed in MALDI. The peaks at *m/z* below 1316.9 indicate the loss of deuterium. Notably, the degree of back exchange was found to be matrix dependent. When CHCA was used as the matrix, the base peak shifted from BTAx6D to BTAx4D (spectrum in blue). Much less, but still significant back exchange was observed when a non‐protic matrix DCTB was used instead of CHCA (spectrum in red). We argue that the matrix dependent behavior of back exchange is due to the different compositions of the MALDI plumes generated from different matrices by laser irradiation. Most likely, the non‐protic DCTB matrix spawned less amount of species with reactive hydrogens that could replace the deuterium labels, thus resulting in less back exchange than CHCA.

Figure 1B shows the MS spectra of a fully deuterated small peptide of angiotensin II (AT) which contains 16 labile hydrogens, all replaced by deuterium. Because DCTB is not a good matrix for the peptide, DHAP was used as another matrix besides CHCA. Here again, serious and matrix‐dependent back exchange in MALDI MS was observed for the peptide.

**FIGURE 2:**
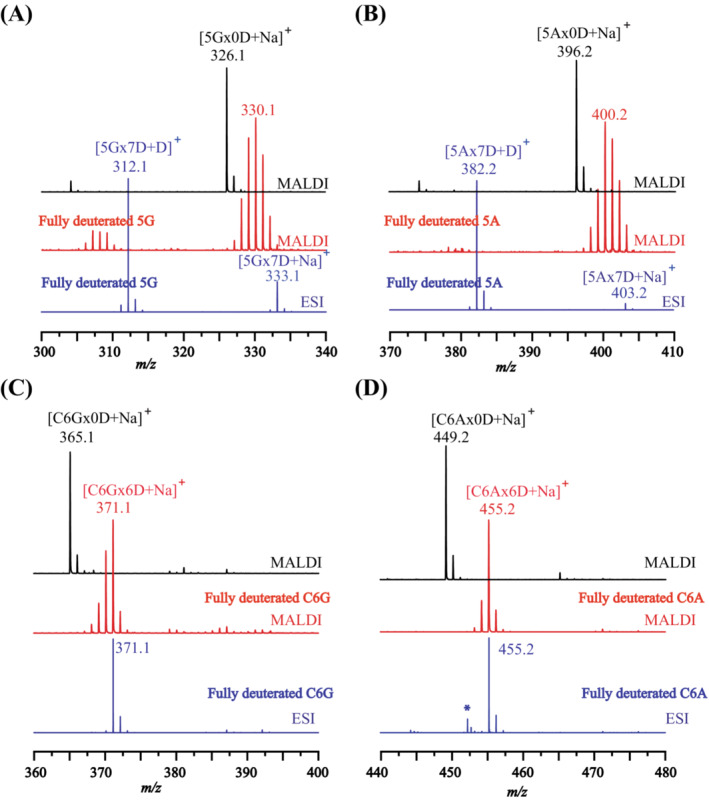
MALDI and ESI mass spectra of pentaglycine (5G), pentaalanine (5A), cyclohexaglycine (C6G), and cyclohexaalanine (C6A). For all the samples, the top (spectrum in black) and bottom (spectrum in blue) spectra are references of the MALDI spectrum of a non‐deuterated sample using CHCA matrix and of the ESI spectrum of the fully deuterated sample, respectively; the middle one is the MALDI spectrum of the fully deuterated sample with CHCA matrix (spectrum in red). (A) Mass spectra for 5G. (B) Mass spectra for 5A. (C) Mass spectra for C6G. (D) Mass spectra for C6A, “*” in the ESI spectrum indicates unknown peaks.

Among the 16 labile deuterium of AT, six are in the backbone amides, seven in the side chains, and three in the end groups. In solution, the labile labels in the side chains and end groups usually have very fast (back) exchange rates, while the labels in the amide backbone show much slower exchange rates which depend on the primary structures of the peptides [1, 2, 3, 4, 5, 6, 28]. To ascertain whether this pattern of exchange rate also holds for the back exchange of deuterated peptides in the MALDI plume, four more small peptides were studied. These peptides are pentaglycine (5G), pentaalanine (5A), cyclohexaglycine (C6G), and cyclohexaalanine (C6A). In contrast with AT, all these four peptides have no side chains. Further, C6G and C6A are cyclic peptides having no end groups at all. The results of these small peptides are presented in Figure 2 and Table 1. In Figure 2, the ESI mass spectra of the fully deuterated peptides and the MALDI TOF mass spectra of the undeuterated peptides are also shown for comparison.

**TABLE 1:** Back exchange values of the fully deuterated peptides tested in MALDI MS with CHCA matrix.^a^, ^b^

	Backbone CONH	Side chain NH OH	End group NH OH	Theoretical average mass of Na^+^ adduct	Measured average mass of Na^+^ adduct	BE%^c^, ^d^
AT	6	7	3	1085.3	1077.8	47 ± 0.56^e^
5G	4	0	3	333.3	329.9	49 ± 1.51
5A	4	0	3	403.4	400.6	40 ± 3.28
C6G	6	0	0	371.3	370.7	10 ± 0.24
C6A	6	0	0	455.5	455.2	5 ± 0.25

^a^ The sample solution and the MALDI plate were cooled down to 0°C with ice prior to sample spotting, and the sample was spotted on top of a preloaded dried layer of CHCA and immediately loaded into the MALDI instrument.

^b^ The BE% values were calculated based on sodium adducts of the peptides.

^c^ Peptides were dissolved respectively in D_2_O and fully deuterated, and the BE% values were calculated by Equation (1).

^d^ The BE% values are likely to be underestimated because of partial deuteration of CHCA matrix by D_2_O (see discussion in the main text).

^e^ Standard deviations based on three measurements.

By comparing Figure 2A with Figure 2C and Figure 2B with Figure 2D, it can be seen clearly that the overall degrees of back exchange (BE%) of the glycine and alanine peptides decrease substantially when the amino and carboxylic acid end groups are eliminated by cyclic reactions. As the peptides were fully deuterated prior to the MALDI experiments, the degrees of back exchange arisen from the MALDI process can easily be calculated by

(1) $$\mathrm{BE}\%=\left(1-\frac{m_{\text{MALDI}}-m_{\text{undeut}}}{m_{\text{deut}}-m_{\text{undeut}}}\right)\times 100\%$$

where *m*_*MALDI*_ is the average mass calculated from the isotopic pattern of a deuterated sample obtained in MALDI, and *m*_*undeut*_ and *m*_*deut*_ the theoretical average molecular masses of the corresponding non‐deuterated and fully‐deuterated peptides, respectively.

From Table 1, it can be seen that the BE% values of the glycine and alanine homo‐peptides dropped from 49% for 5G to 10% for C6G, and from 40% for 5A to 5% for C6A, respectively. The results, together with the high BE% of AT, strongly indicate that the pattern of back exchange in the MALDI plume is similar with that in solution, that is, the back exchange reactions in the end groups and side chains are much faster than those in the back bone amides. Further, the back exchange of C6A is about two folds less than C6G. As the C6A and C6G have the same number of amide in the backbones, it seems that deuterium labels were of some sort protected by the side chain of alanine.

**FIGURE 3:**
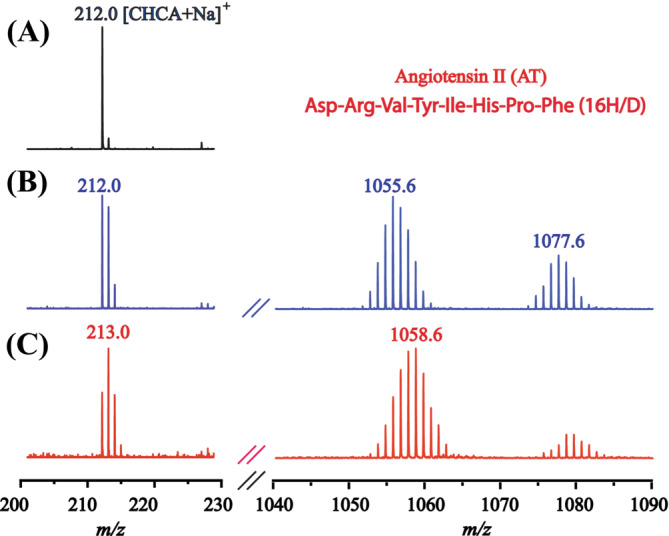
MALDI TOF mass spectra of CHCA and angiotensin II (AT) with different lengths of contact time of D_2_O with matrix. (A) CHCA matrix without contact with D_2_O, (B) the target plate loaded immediately into the MALDI vacuum after the D_2_O solution was spotted on top of a preloaded layer of matrix, and (C) the target plate loaded into the MALDI vacuum 5 min after the D_2_O solution was spotted on top of a preloaded layer of matrix.

In all the experiments presented above, fully deuterated samples in D_2_O were measured. Although the matrices tested here are not soluble in D_2_O, the contact with D_2_O can still make the matrices (partly) deuterated. Figure 3 shows the mass spectra of AT with different lengths of contact time between CHCA and D_2_O. The mass spectrum of CHCA without deuteration is also shown in this figure for comparison. In Figure 3B, the target plate was immediately loaded into the MALDI vacuum after the D_2_O solution was spotted on top of a preloaded layer of matrix, while in Figure 3C, the plate was loaded 5 min later after sample spotting. The spectrum shown in Figure 3B indicates that significant amount of CHCA was deuterated after only a very brief contact with D_2_O. Evidently, less back exchange would occur if more matrix molecules were deuterated. As expected, the longer contact time in Figure 3C led more matrix molecules to be deuterated, and thus, less back exchange was observed for AT. It can be argued that even a forward exchange might be anticipated in HDX MS of real samples if the matrix is highly deuterated. For a reliable measurement, however, it is vital that any changes to the amount of labels after labelling must be minimized. The labels should not disappear (back exchange) nor should more labeling occur (forward‐exchange) during the analysis [1, 5]. Unfortunately, this requirement is difficult to satisfy because of the very reactive environment in the MALDI plume. If a deuterated solvent is used for the preparation of the MALDI spot, the (partially) deuteration of the matrix could become an additional problematic variable that will affect the degree of back exchange. As discussed above, back exchange or even forward exchange can occur in MALDI, depending on the experimental conditions. Therefore, extra care must be taken during sample preparation and in the interpretation of the obtained data when MALDI is employed for the HDX MS analysis.

As CHCA is insoluble in cold D_2_O, deuteration of the matrix must be due to the wetting by a large excess of D_2_O. It should be noted here that before laser irradiation, the wetting of matrix will not cause considerable extra back exchanges to the analytes. After depositing a sample solution on the matrix surface, the possible influence of matrix on the deuterium level prior to laser irradiation can be divided into two parts: (1) before and (2) after complete solvent evaporation. For the 1st part, as CHCA is insoluble in cold water, the influence of matrix on the deuterium level in the sample solution should be minimal. Taking a step back, even if the matrix were completely dissolved by cold water, the matrix concentration would be only about 1% (about 10 μg preloaded matrix and 1 μL D_2_O solution). Furthermore, the MALDI experiments discussed above for back exchange were performed with D_2_O. If the matrix were dissolved by D_2_O, it would be fully deuterated and would not cause back exchange. For the 2nd part after solvent evaporation, the sample is on top of the preloaded matrix layer. We did not observe continued back exchange in the dried sample as no considerable changes were observed after leaving the dried sample in the MALDI chamber for 3 h. This is logical because reactions between compounds in two separate solid layers, with one on top of the other, are slow.

Also, the degree of back exchange will depend on the chosen instrument and its settings. In MALDI measurements, delayed extractions (DE) are usually used to improve the mass resolution. In this work, the delay time is set at 130 ns for good mass resolution. Although a decreased DE time will shorten the collision/reaction time in the MALDI plume, and thus, reduce the degree of back exchange, it could significantly lower the resolution as well. It should be noted here that the artificial back exchange cannot be prevented even if DE = 0, because collisions between ions and the reactive species in the dense plumes will certainly continue during the extraction of ions. Furthermore, the exact time at which ions are formed are difficult to predict [21]. Ions might have already been formed via various routes in the plume, including the routes involving back exchange reactions, even for the extraction without delay (DE = 0).

In the paragraphs above, the overall back exchange of fully deuterated peptides was discussed. In real applications, on the other hand, the peptides are generated by digestion of proteins followed by HPLC separation. In order to preserve the deuterium labels, digestion and HPLC separation are carried out at quenched conditions. Under these conditions, deuterium labels in the side chains and end groups are replaced back to hydrogen while the labels in the backbone amides can mostly be preserved. As the most important information of HDX are stored in the backbone amides, the loss of D‐labels in the sidechains and end groups is not a problem and might even be beneficial for simplifying the MS analysis because it removes a large deuteration background [29]. In addition to the labels in the sidechains and end groups, however, the backbone amide of N‐terminal will undergo quick back exchange as well [6, 30, 31]. Depending on the experimental conditions, 10%–30% of the deuterium labels in the backbone amides might be lost during the digestion and HPLC separation [3].

**FIGURE 4:**
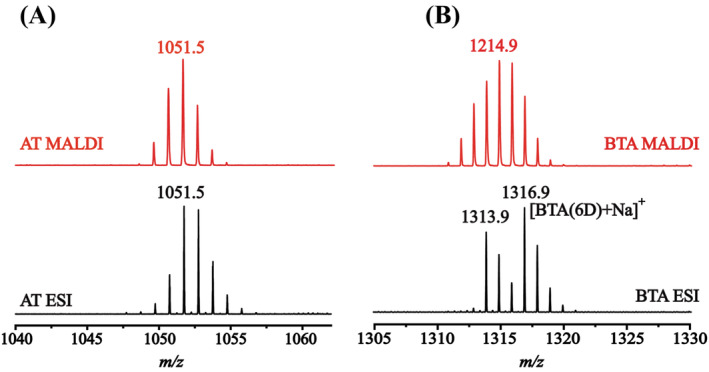
MALDI and ESI mass spectra of deuterated angiotensin II (AT) under quenched conditions and of BTA after HDX for 2 h. (A) Mass spectra for deuterated AT, 0.1 mL of a fully deuterated peptide solution was first cooled down to 0°C and then added into 2 mL of a quenching solution (0.1% formic acid in H_2_O at 0°C). The quenched solution was equilibrated for 2 min before being measured by ESI MS (bottom spectrum in black) or MALDI MS with CHCA matrix (top spectrum in red). (B) A BTA solution in H_2_O was diluted 100 times in D_2_O, exchanged for 2 h before being measured by ESI MS (bottom spectrum in black) or MALDI MS with DCTB matrix (top spectrum in red).

To study the back exchange on the backbone amides in MALDI after the D‐labels in the side chains and end‐groups had all been reverted back to H, the fully deuterated AT sample was diluted 20 times in H_2_O under quenched conditions. The resultant MALDI and ESI mass spectra are shown in Figure 4A. Because of the back exchange reactions in the MALDI plume, the peptide ion peaks in the MALDI spectrum are shifted to lower mass regions when compared to the corresponding ESI spectrum. The theoretical average molecular mass of non‐deuterated [AT + H]^+^ is 1047.2. In Figure 4A, the average mass obtained from MALDI and ESI are 1051.4 and 1052.0, which indicates there are 4.2 and 4.8 amide D‐labels left in the MALDI and ESI measurements, respectively. Assuming that the back exchange in ESI is negligible, the loss of the D‐labels in the backbone amides of AT is estimated to be around 13% in MALDI relative to ESI. As MALDI is very often used directly without HPLC separation [3, 12], the loss of deuterium labels in the MALDI plume could largely be compensated for by skipping the HPLC step. Moreover, in many cases where the biological functional information about the location of folding/unfolding is of primary interest, certain degree of back exchange can be tolerated because the actual number of deuterons is not as important as where the variation of exchange has occurred under different conditions [1, 2, 3, 4, 5, 6]. Thus, the back exchange caused by the MALDI process should not be considered to be an insurmountable obstacle hindering the application of MALDI.

However, there are also circumstances where the back exchange occurred inside the MALDI plume can indeed be a serious problem in the HDX MS analysis. Figure 4B shows the MALDI and ESI mass spectra of BTA supramolecular polymer after HDX for 2 h. Since the formation of supramolecular polymers in water relies on the combination of non‐covalent bonds and hydrophobic interactions, solvent evaporation in the ion source will induce the breakdown of the polymers into monomers. Therefore, the steps of digestion and subsequent HPLC separation, that can cause back exchange but are often necessary for the analysis of the protein samples using ESI MS, are not required for such supramolecular polymers. In other words, the HDX solution can directly be introduced into the ESI source. In the ESI spectrum, two distinct distributions of BTAx3D and BTAx6D were observed. The BTA unit has three hydroxyl hydrogens at the tetra (ethylene glycol) peripheries and three amide hydrogens at the core. For the BTA supramolecular fibers, the three peripheral hydrogen atoms in the OH groups are immediately exchanged for deuterium atoms because of their constant accessibility to the solvent, while the three NH groups are located in the hydrophobic core and are protected from solvent [25]. BTAx6D is formed over time as also the three labile NH hydrogen atoms get exchanged into NDs. The isotopic pattern for BTAx3D is the result of the rapid exchange of the hydrogens of the OH groups for deuterium, and the pattern for BTAx6D is the result of HDX of both the OH and the NH groups. By taking the isotopic distribution of BTAx3D and trace amount of H_2_O into account, BTAx4D and BTAx5D are absent in the ESI spectrum, which explicitly indicates the monomer exchange mechanism of the supramolecular polymer [7]. In contrast, this EX1‐like distribution pattern becomes obscured in the MALDI spectrum. The species of BTAx4D and BTAx5D which are absent in the ESI spectrum are prominently present in the MALDI spectrum. Apparently, the otherwise nonexistent species of BTAx4D and BTAx5D were generated from BTAx6D because of the back exchange in the MALDI plume.

In conclusion, we have clearly demonstrated that the reactive MALDI plume can cause substantial back exchange in HDX MS analysis. The back exchange in MALDI plume follows a similar pattern as in solution, that is, the labels in the side chains and end groups exchange at much higher rates than the labels in the amide backbones. For the peptides tested, 5%–15% of deuterons in the backbones were lost during the MALDI process. This degree of back exchange is comparable with that occurred during HPLC separation, and should be tolerable for most HDX MS applications. However, it is also important to be aware that the back exchange could seriously trouble the analysis of supramolecular polymers and samples with EX1 mechanism because the distinct isotopic distributions would become blurred or even vanished due to the back exchange. It should be emphasized here that the aim of this application note is not to discourage the use of MALDI in HDX MS, but rather to remind users that considerable back exchange could occur inside the reactive MALDI plume.

## Data Availability

The data that support the findings of this study are available on request from the corresponding author. The data are not publicly available due to privacy or ethical restrictions.
